# Editorial Comment: Female Urethroplasty: A Critical Review of Indications, Techniques and Concerns

**DOI:** 10.1590/S1677-5538.IBJU.2026.9914

**Published:** 2026-07-08

**Authors:** Luciano A. Favorito

**Affiliations:** 1 Universidade do Estado do Rio de Janeiro Unidade de Pesquisa Urogenital Rio de Janeiro RJ Brasil Unidade de Pesquisa Urogenital - Universidade do Estado do Rio de Janeiro - Uerj, Rio de Janeiro, RJ, Brasil; 2 Hospital Federal da Lagoa Serviço de Urologia Rio de Janeiro RJ Brasil Serviço de Urologia, Hospital Federal da Lagoa, Rio de Janeiro, RJ, Brasil

**Joy Narayan Chakraborty ^1^, Bhavatej Enganti ^2^, Prashant Nayak ^3^**

*^1^ Apollo Hospital, Guwahati, India; ^2^ Asian Institute of Urology, Hyderabad, India; ^3^ Department of Urology, AIIMS, Bhubaneswar, India*

**Int Urogynecol J. 2026 Apr;37(4):823-833**

DOI: 10.1007/s00192-025-06415-4 | ACCESS: 41204975

## COMMENT

Urethral stricture is one of the most challenging conditions in reconstructive surgery (1-4). Many techniques have been described and there is extensive experience regarding the treatment of urethral strictures in men; however, urethral strictures in women are rarer and are the subject of far fewer publications. In tis paper of Chakraborty and collegues (5) we can observe a very nice narrative review about the indications, techniques and concerns of female urethroplasty. The authors shows a practical guide for urologists managing female urethral strictures, including an overview of the female urethral sphincter mechanism and the most common urethroplasty techniques. Institutional ethics board approval is not required for this review article; however, proper consent for using operative images involving human participants has been obtained from the respective patients before surgery. We congratulate the authors for the interesting paper.

## Data Availability

Data Not Provided
